# Effectiveness of continuous glucose monitoring during diabetic pregnancy (GlucoMOMS trial); a randomised controlled trial

**DOI:** 10.1186/1471-2393-12-164

**Published:** 2012-12-27

**Authors:** Daphne N Voormolen, J Hans DeVries, Arie Franx, Ben WJ Mol, Inge M Evers

**Affiliations:** 1 Department of Obstetrics and Gynaecology, University Medical Centre Utrecht, Utrecht, the Netherlands; 2 Department of Endocrinology, Academic Medical Centre Amsterdam, Amsterdam, the Netherlands; 3 Department of Obstetrics and Gynaecology, Academic Medical Centre Amsterdam, Amsterdam, the Netherlands; 4 Department of Obstetrics and Gynaecology, Maxima Medical Centre Veldhoven, Veldhoven, the Netherlands; 5 Department of Obstetrics and Gynaecology, Meander Medical Centre Amersfoort, Amersfoort, the Netherlands

**Keywords:** Diabetes, Pregnancy, Continuous glucose monitor, Macrosomia, Effectiveness

## Abstract

**Background:**

Hyperglycemia in pregnancy is associated with poor perinatal outcome. Even if pregnant women with diabetes are monitored according to current guidelines, they do much worse than their normoglycaemic counterparts, marked by increased risks of pre-eclampsia, macrosomia, and caesarean section amongst others. Continuous Glucose Monitoring (CGM) is a new method providing detailed information on daily fluctuations, used to optimize glucose control. Whether this tool improves pregnancy outcome remains unclear. In the present protocol, we aim to assess the effect of CGM use in diabetic pregnancies on pregnancy outcome.

**Methods/design:**

The GlucoMOMS trial is a multicenter open label randomized clinical trial with a decision and cost-effectiveness study alongside. Pregnant women aged 18 and over with either diabetes mellitus type 1 or 2 on insulin therapy or with gestational diabetes requiring insulin therapy before 30 weeks of gestation will be asked to participate. Consenting women will be randomly allocated to either usual care or complementary CGM. All women will determine their glycaemic control by self-monitoring of blood glucose levels and HbA1c. In addition, women allocated to CGM will use it for 5–7 days every six weeks. Based on their CGM profiles they receive dietary advice and insulin therapy adjustments if necessary. The primary outcome measure is rate of macrosomia, defined as a birth weight above the 90th centile. Secondary outcome measures will be birth weight, composite neonatal morbidity, maternal outcome and costs. The analyses will be according to the intention to treat principle.

**Discussion:**

With this trial we aim at clarifying whether the CGM improves pregnancy outcome when used during diabetic pregnancies.

**Trial registration:**

Nederlands Trial Register: NTR2996

## Background

Diabetes during pregnancy is a high risk situation for both mother and the child. Optimising glycaemic control is a key feature of prenatal care for diabetic women [1]. Since the 1960s, there has been a reduction in perinatal morbidity and mortality [2,3]. However, even if pregnant women with diabetes are monitored according to current guidelines, they do much worse than their normoglycaemic counterparts. In a nationwide study in 2000 on the outcome of 323 women with a pregnancy complicated by type 1 diabetes we found a high prevalence of maternal, perinatal and neonatal complications [4]. These complications occurred despite good pre-pregnancy care, as 84% of these pregnancies were planned and 70% used adequate folic acid supplementation. Overall, glycaemic control during these pregnancies was acceptable, as average HbA1c value was 44 mmol/mol. Nevertheless, these pregnancies showed high complications rates that require improvement.

The continuous glucose monitoring system (CGMS) is a new technique that retrospectively provides detailed information regarding glucose fluctuations during the day. The CGMS has been studied in non-pregnant patients where it has demonstrated clinical usefulness by enhancing decision-making through detecting previously unrecognised postprandial hyperglycaemia and nocturnal hyper-and hypoglycemia [5]. Scientific evidence on a HbA1c-reducing effect of CGMS use is limited [6,7]. Although some studies do evaluate the effect of CGMS use on biochemical endpoints, such as HbA1c levels, data on clinical endpoints like diabetic complications, are lacking. The usefulness of CGMS use during pregnancy has hardly been evaluated up to now [7]. A recent RCT showed that intermittent CGMS use during pregnancy in 71 women with pre-existing diabetes resulted in a significant reduction in HbA1c at 32–36 weeks gestation. Furthermore, the odds ratio for reduced risk of macrosomia was 0.36 (95% CI 0.13-0.98, p = 0.05) [8]. However, this study was hampered by small sample size and both study groups differed in composition (e.g. 5 set of twins in the intervention group as opposed to none in the control group). Furthermore 11 (4%) of the children in the intervention group were small for gestational age (≤10 centile) as opposed to none in the control group. This difference did not reach statistical significance either but may point to an adverse effect of CGMS. Another recent RCT by Secher et al. investigated the effect of intermittent real time CGMS use during pregnancy in 154 women with pre-existing diabetes on pregnancy outcome [9]. The rate of macrosomia was not significantly different between the intervention group (43%) and the control group (30%) (p = 0.09). Also, HbA1c levels were similar in both groups. The investigators concluded that intermittent CGMS use did not improve glycaemic control in pregnancy nor did it improve pregnancy outcome. Thus, further evaluation in larger studies is urgently needed before wide implementation of CGMS during pregnancy.

### Relevance

Despite improvements in blood glucose monitoring technology, and obstetric and neonatal care over the last decades, maternal and fetal complications occur much more frequent in diabetic women than in non-diabetic women [4]. Our Dutch nationwide study of type 1 diabetes mellitus in pregnancy showed high complication rates in diabetes pregnancies. Maternal complications were episodes of severe hypoglycemia (40%), pre-eclampsia (13%) and caesarean section (44%). Perinatal and neonatal complications included congenital malformations (9%), prematurity (32%), perinatal mortality (3%), macrosomia (45%) and neonatal morbidity such as shoulder dystocia (14%), all remarkably higher rates than those in non-diabetic pregnancies [4]. Similar rates of complications have recently been found in other nationwide studies in Denmark, the United Kingdom and Sweden [10-12]. In the Dutch study 45% of the newborns were macrosomic and 24% were extremely macrosomic (>p97.7). Recent data from Denmark show that 56% of the newborns of type 2 diabetic women were macrosomic [11].

The prevalence of gestational diabetes is increasing and comprises approximately 5% of the pregnant women. Given the worldwide rising incidence of diabetes, as a consequence of changed life style and consequent obesity, improvement of obstetric care for diabetic patients is essential [13,14]. Moreover, it has been shown that children born macrosomic are at risk for developing obesity and diabetes mellitus type 2 at a young age [15-17]. Reduction of macrosomia will not only reduce the risk of perinatal complications but may also prevent these future health problems.

## Methods/design

### Aims

This trial evaluates the clinical effectiveness, costs and cost-effectiveness of CGMS use with the aim to optimize glycaemic control and pregnancy outcome of diabetic pregnancies relative to standard control methods. The primary outcome measure is macrosomia. Secondary obstetric outcome measures are birth weight, neonatal and maternal morbidity. Furthermore, diabetic outcome measures are HbA1c level, and glucose variability. In addition, cost-effectiveness will also be evaluated.

### Study design

The study will be a multicentre randomised controlled trial comparing standard care to standard care with additional CGMS use. The study will be open, as it is impossible to blind the pregnant women and health care workers involved for the strategy to which the women are allocated. This trial is performed by the Dutch Obstetric Consortium, a collaboration of the majority of hospitals in the Netherlands. It supplies research nurses and online services, and is supported by the Dutch Society of Obstetrics and Gynaecology. Approximately 25 hospitals, including university hospitals, teaching hospitals and non-teaching hospitals will participate in this trial.

The study has been approved by the ethics committee of the Academic Medical Centre Amsterdam (reference number 10/322 # 11.17.0554) and in addition by the boards of management of all participating hospitals.

### Participants

Pregnant women with pre-existing diabetes mellitus (type 1 or 2) before gestational age of 16 weeks or with gestational diabetes requiring insulin therapy before 30 weeks gestational age will be asked to participate. Inclusion criteria include maternal age of at least 18 years and need for insulin treatment by means of injections or insulin pump. Patients with severe medical or psychological comorbidity will be excluded. Multiple pregnancies will also be excluded since they require different standards of obstetric evaluation.

### Recruitment and randomisation

Potential study candidates will be identified by their gynaecologist or internist. Patients eligible for participation in the study will be invited for additional counselling by a research nurse, to ensure that they will be fully informed on the nature of the study by means of both oral and written information. Patients who agree to participate will be asked to sign a written informed consent of which they will receive a copy.

After providing informed consent for the participation in the study, the patient will be randomly allocated to either standard care or standard care and additional CGMS use. Randomisation will be done on a 1:1 basis over the internet, stratified for type of diabetes, using a web-based program. The current inclusion status is being displayed on the GlucoMOMS trial website http://www.studies-obsgyn.nl/glucomoms/page.asp?page_id=1027

### Hypothesis

The CGMS has been shown to potentially improve glycaemic control as defined by HbA1c levels. Since obstetric complications in diabetic pregnancies, especially macrosomia, seem to be related to glycaemic control, we hypothesise that the additional use of CGMS will reduce the macrosomia rate by 30 percent.

### Intervention

The continuous glucose monitoring system (CGMS) is a new technique that is efficacious in the monitoring of diabetic women. It can provide a comprehensive glucose profile over a 5–7 days period. The CGMS (CGMS Medtronic: Minimed, Northridge,CA) measures glucose levels through electro-chemical detection in the extracellular fluid of the abdominal subcutaneous tissue and stores values in a range of 2.2 - 22.2 mmol/L every 10 sec. An average value is stored in the monitor every 5 minutes, providing up to 288 blood glucose measurements (12x24) a day. The subjects are unaware of the results of the sensor measurements during monitoring and need to continue self-monitoring of blood glucose while carrying the monitor. After 5–7 days glucose profiles are obtained from the monitor and can be evaluated by the diabetologist and dietary or insulin therapy changes can be advised.

**Figure 1:**
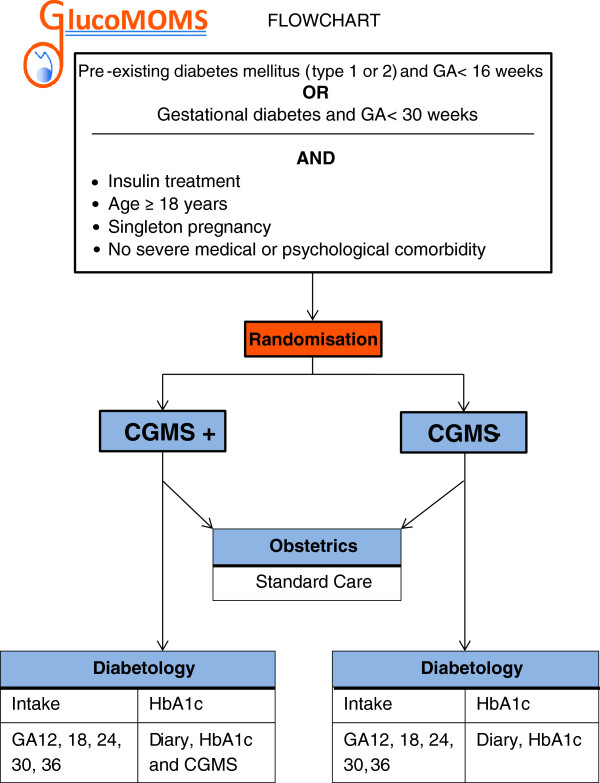
Flowchart.

All study participants will determine their glycaemic control by self-monitoring of blood glucose levels (4–8 times/day) and HbA1c-levels (every 4 weeks) throughout the course of pregnancy. Figure 1 represents a flowchart of the study.

### Outcome measures

#### Main study parameter/endpoint

Outcome measures of the trial are related to obstetric complications and glycaemic control. Macrosomia is the most frequent complication of diabetes during pregnancy and is associated with neonatal morbidity and long term effects. Therefore, the primary outcome measure is macrosomia rate. Macrosomia is defined as a birth weight above the 90^th^ centile.

Secondary maternal endpoints are: pre-eclampsia (defined as systolic blood pressure ≥ 140 mmHg or diastolic blood pressure ≥ 90 mmHg occurring after 20 weeks of gestation and simultaneous proteinuria of ≥300 mg/24 hours) caesarean section, hypoglycemia (subdivided as biochemical hypoglycemia (self-measured blood glucose < 3.9 mmol/l) or symptomatic hypoglycemia (symptoms of hypoglycemia confirmed by self-measured blood glucose < 3.9 mmol/l) or severe hypoglycemia, (prompting the need for help by another person)), HbA1c levels throughout the course of pregnancy, glucose variability (Mean Absolute Glucose change per patient per hour) and relative glucose variability (Coefficient of Variability) [18].

Secondary neonatal endpoints are: birth weight, preterm birth, perinatal death, birth trauma (including shoulderdystocia, clavicle fracture or Erb’s palsy), neonatal hypoglycemia (defined as blood glucose < 2.6 mmol/l), respiratory distress syndrome (RDS), broncho pulmonal dysplasia (BPD), intraventricular haemorrhage II B or worse, necrotizing enterocolitis (NEC), and sepsis.

#### Follow up

Long term follow up of the offspring is desirable as long term effects of diabetic pregnancy on infants, in particular those born macrosomic, have become evident. Long term follow up is planned but will depend on future funding.

#### Economic evaluation / cost analysis

With two or three continuous glucose monitors (hardware), costing about €1,500 each, the total monitor costs are €37,500 per centre. We will use 5 sensors (each €50) per pregnancy, resulting in sensor costs of €37,500, bringing total equipment costs at €75,000. The additional costs of CGMS per year (per pregnancy) are €500. With these crude estimates, a hypothesized 33% reduction in macrosomia rate, would imply for the short term a cost-effectiveness ratio of €1,500 per prevented case of macrosomia, €6,076 per prevented caesarean section, €15,189 per prevented case of preterm birth, and €48,605 per prevented case of neonatal death. A strong reduction of macrosomia would also reduce downstream resource use and associated costs of neonatal complications. The question is to what extent downstream economic benefits also offset the costs required to obtain these clinical outcomes, and whether the resulting cost-to-benefit ratio justifies a standard policy of using CGMS during pregnancy [19,20].

Rising health care costs and reimbursement of expensive medical devices are not justified unless extensive cost-effective analysis turns out favourable. Therefore, besides evaluation of the effectiveness of CGMS on pregnancy outcome, resource utilization and costs for maternal and neonatal care will be analysed as well.

### Statistics

#### Sample size

We anticipate that a reduction of macrosomia from 45% to 30% will outweigh the costs of the additional use of the CGMS. As we assume 10% protocol violations and drop out, we need to randomise 300 women (Alpha-error .05, Beta-error .20, one-sided test) of which 150 will receive additional CGMS use and 150 will be controls.

#### Data analysis

Data will initially be analysed according to the intention to treat method. The main outcome variable, macrosomia, will be assessed by calculating rates in the two groups, relative risks and 95% confidence intervals as well as numbers needed to treat. The secondary outcome measures will be addressed in a similar manner. Continuous outcome measures will be compared using parametric and non-parametric tests, depending on the distribution of the data. We plan a subgroup analysis of women with diabetes type 1 and type 2 diabetes and gestational diabetes.

#### Interim analysis

An interim analysis will be performed after the inclusion of 150 women. This analysis will be done by an independent data and safety monitoring committee, which will not be aware of the allocation of sensor or routine control when they judge data on effectiveness. In case of strong effects, the safety monitoring committee can advise to stop the study. Furthermore, each SAE will be reported to the data and safety monitoring committee.

## Discussion

Women with diabetes in pregnancy are at high risk for unfavourable pregnancy outcomes. Strict glycaemic control is required during pregnancy to minimize complications. After its introduction, continuous glucose monitoring has gained ground quickly in diabetes care in order to optimize glycaemic control. However, the clinical and cost-effectiveness of CGMS to improve pregnancy outcome remains to be clarified. This randomised controlled trial aims to evaluate the effect of CGMS use on pregnancy outcome in diabetic pregnancies.

## Abbreviations

GA: Gestational age; CGM: Continuous glucose monitoring; CGMS: Continuous glucose monitoring system; RCT: Randomised controlled trial; CI: Confidence interval; RDS: Respiratory distress syndrome; BPD: Broncho pulmonal dysplasia; NEC: Necrotizing enterocolitis.

## Competing interests

The authors declare that they have no competing interests.

## Authors’ contributions

IME, AF and BWM were involved in conception and design of the study. JHD has made substantial contributions to the design and conduction of the study. DNV, JHD, AF, BWM and IE drafted the manuscript. All authors mentioned in the manuscript are members of the GlucoMOMS-trial study group. All authors read, edited and approved the final manuscript.

## Pre-publication history

The pre-publication history for this paper can be accessed here:

http://www.biomedcentral.com/1471-2393/12/164/prepub
